# A Case of Enteritis, with Obstruction of Intestine

**Published:** 1863-11

**Authors:** 

**Affiliations:** New Salem, Fairfield Co., Ohio; New Salem, Fairfield Co., Ohio


### A CASE OF ENTERITIS—WITH OBSTRUCTION OF INTESTINE.

Reported by Drs. YOUNTZ and GOSS,

*Of New Salem, Fairfield Co., Ohio,*

AND COMMUNICATED WITH REMARKS BY

TOM. O. EDWARDS, M. D., of Lancaster, Ohio.

Was called to see Mrs. Kagy, Oct. 12th, and found her vomiting a bilious fluid, complaining of great pain over the umbilical region, slight tympanitis, with furred tongue, and pulse contracted. Gave her 3 grs. Sub. Mur. Hydr. with ½ gr. Sulph. Morphine, every 3 hours, and ordered sinapism to stomach, to be followed with hop poultice.

13th.—No improvement; patient still vomiting at intervals of 3 hours; bowels more tympanitic; pain more intense, and no action on bowels. Gave her 7 compound Cathartic pills and ordered a dose of oil and turpentine if no action in 6 hours—continued sinapism and fomentations

14th.—No amelioration of the symptoms; bowels more swollen; some difficulty in urinating; vomiting continues as before; great tenderness over the entire abdomen. Gave her Hyd. Sub. Mur., grs. v; Dover's, grs. iij; every 3 hours, and ordered the bowels distended with enemas every hour.

15th.—Found symptoms all aggravated; vomiting purely stercoraceous matter. Gave her croton oil, 3 drops every hour, until she had taken ten portions; also continued fomentations with enema of oil and turpentine.

16th.—No improvement in the case. Stercoraceous vomiting still continues. Abdomen very hard and distended. Countenance still good, with very fair circulation, and skin natural. Left her on Sub. Mur., grs. x; Opium, grs. ij; every 3 hours. Ordered sinapisms to be followed with hop poultice, and continued the use of enemata composed of castor oil, turpentine and milk, every hour.

17th.—Met Dr. Tom. O. Edwards. Ordered Cal., grs. 20; Opii, grs. iij; every three hours. Epispastic over whole abdomen.

18th.—Met Drs. Edwards and Prof. Blackman, who was in Lancaster.

19th.—No change in symptoms. Same treatment continued. Stercoraceous vomiting once in 26 hours. Well controlled.

20th.—Found patient very restless, constantly tossing about in bed, with manifest anxiety of countenance. Still no sleep. Increased Opium to grs. vj.

21st.—Found patient very listless and languid; extremities cold; small pulse, with great anxiety of countenance; refuses to take any more medicine, but vomiting stercoraceous matter, finally prevailed upon her to take some. Continued use of Calomel and Opium; vomiting freely of same matter. Found stricture in rectum forbidding introduction of the enema pipe —overcome by finger.

22nd.—No change since yesterday. Gave her 15 grains of alum every 3 hours, and ordered fomentations over blistered surface continued.

23rd.—Found the tympanitis subsiding, countenance more hopeful; extremities warm, but still no action on bowels; vomiting ceased. Saw her again at 9 P. M., said she felt like having an action on bowels. Distended bowels freely with tepid water enema, which was followed in about ten minutes with a very free discharge, consisting mostly of Scybala. Repeated the enema in about fifteen minutes with like result—after which the distension of the bowels subsided rapidly; the patient felt easy and sunk into a fine calm sleep. Ordered the hop poultice continued, and left her for the night.

24th—Found our patient easy and comfortable—told us that she had two more motions of the bowels attended with a very unpleasant smell. Complained of slight pain in bowels; tongue red at tip. Ordered a dose of oil with a teaspoonful of turpentine, and the hot fomentations continued.

25th.—Found Mrs. K. sitting in an arm-chair feeling very comfortable, having just eaten a very respectable breakfast. She had three operations on the bowels during the preceding night. Ordered nothing but quiet.

26th.—Dismissed the case for want of any further indications for treatment.

Remarks.—Did the alum act on the contractility of the muscular coat of the intestines—or was this a *post hoc ergo propter hoc?* Enemas were most industriously used by aid of pipes reaching far up in the bowel. Drs. Yountz and Goss deserve great credit for their pertinacity and attention to this case. This is the second attack—the first yielding in four days—from what cause is not known. I regard the persistent use of Opii as the most successful in these cases. Yet the alum (most opportune in Cholica Pictonum and in the so-called Milk Sickness) where we have fatal distension of the bowels from the poison. The patient is now well and bore her severe affliction with much fortitude.

Tom. O. Edwards.

Lancaster, Ohio, Oct. 30th, 1863.

